# Post-transfusion activation of coagulation pathways during severe COVID-19 correlates with COVID-19 convalescent plasma antibody profiles

**DOI:** 10.1172/JCI181136

**Published:** 2025-03-17

**Authors:** Svenja Weiss, Hung-Mo Lin, Eric Acosta, Natalia L. Komarova, Ping Chen, Dominik Wodarz, Ian Baine, Ralf Duerr, Ania Wajnberg, Adrian Gervais, Paul Bastard, Jean-Laurent Casanova, Suzanne A. Arinsburg, Talia H. Swartz, Judith A. Aberg, Nicole M. Bouvier, Sean T.H. Liu, Raymond A. Alvarez, Benjamin K. Chen

**Affiliations:** 1 Division of Infectious Diseases and; 2 Department of Population Health Science and Policy, Icahn School of Medicine at Mount Sinai, New York, New York, USA.; 3 Department of Mathematics and; 4 Department of Ecology, Behavior and Evolution, UCSD, La Jolla, California, USA.; 5 Department of Transfusion Medicine, Icahn School of Medicine at Mount Sinai, New York, New York, USA.; 6 Department of Medicine,; 7 Department of Microbiology, and; 8 Vaccine Center, NYU Grossman School of Medicine, New York, New York, USA.; 9 Division of General Internal Medicine, Icahn School of Medicine at Mount Sinai, New York, New York, USA.; 10 St. Giles Laboratory of Human Genetics of Infectious Diseases, The Rockefeller University, New York, New York, USA.; 11 Laboratory of Human Genetics of Infectious Diseases, INSERM, Necker Hospital for Sick Children, Paris, France.; 12 Imagine Institute, University of Paris, Paris, France.; 13 Howard Hughes Medical Institute, New York, New York, USA.; 14 Department of Pathology and; 15 Department of Microbiology, Icahn School of Medicine at Mount Sinai, New York, New York, USA.

**Keywords:** COVID-19, Immunology, Adaptive immunity, Immunoglobulins, Immunotherapy

## Abstract

Early antibody therapy can prevent severe SARS-CoV-2 infection (COVID-19). However, the effectiveness of COVID-19 convalescent plasma (CCP) therapy in treating severe COVID-19 remains inconclusive. To test a hypothesis that some CCP units are associated with a coagulopathy hazard in severe disease that offsets its benefits, we tracked 304 CCP units administered to 414 hospitalized COVID-19 patients to assess their association with the onset of unfavorable post-transfusion D-dimer trends. CCP recipients with increasing or persistently elevated D-dimer trajectories after transfusion experienced higher mortality than those whose D-dimer levels were persistently low or decreasing after transfusion. Within the CCP donor-recipient network, recipients with increasing or persistently high D-dimer trajectories were skewed toward association with a minority of CCP units. In in vitro assays, CCP from “higher-risk” units had higher cross-reactivity with the spike protein of human seasonal betacoronavirus OC43. “Higher-risk” CCP units also mediated greater Fcγ receptor IIa signaling against cells expressing SARS-CoV-2 spike compared with “lower-risk” units. This study finds that post-transfusion activation of coagulation pathways during severe COVID-19 is associated with specific CCP antibody profiles and supports a potential mechanism of immune complex–activated coagulopathy.

## Abstract 2

Analysis of a large donor-recipient convalescent plasma network identifies high-risk plasma associated with coagulopathy in multiple recipients carrying antibodies with distinct pro-inflammatory profiles.

## Graphical abstract

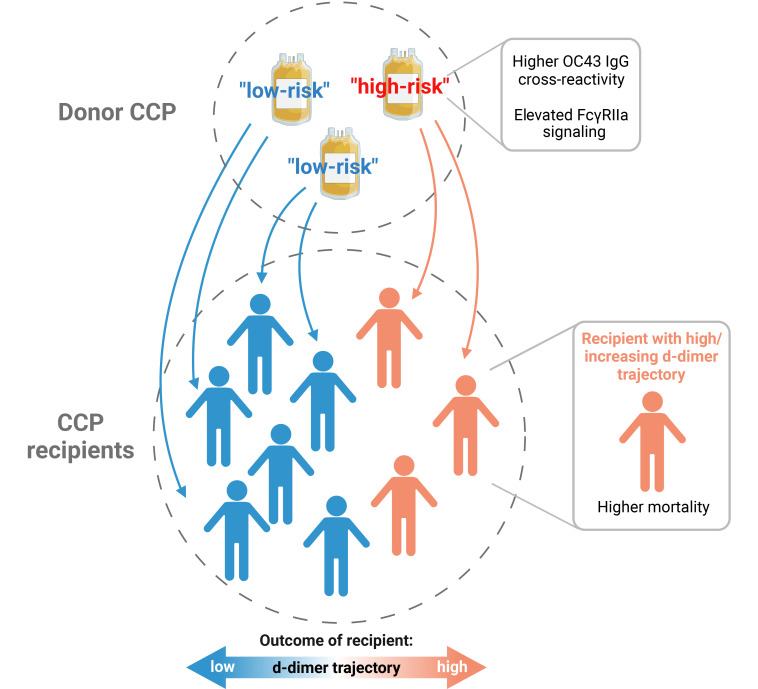

## Introduction

During the COVID-19 pandemic, numerous randomized controlled trials of convalescent plasma were conducted to determine its efficacy. Initial retrospective observational studies in 2020 suggested that COVID-19 convalescent plasma (CCP) was beneficial for hospitalized COVID-19 patients (1–3), especially with high-titer antibodies (1). However, randomized controlled trials (RCT) and meta-analyses have not demonstrated clear benefit for hospitalized COVID-19 patients (4–8), except in specific populations (e.g., immunocompromised) (7, 9). While numerous studies have not found CCP to be associated with increased hazard (1, 10, 11), they have not explicitly addressed the donor heterogeneity of CCP that can have different abilities to mediate positive antiviral or negative, potentially overexuberant inflammatory responses. Therefore, we set out to examine whether the apparent lack of efficacy of CCP seen in RCTs is because CCP does not confer any clinical benefit in hospitalized COVID-19 or whether some clinical benefits are offset by hidden harmful effects from some CCP donor subgroups (12).

Numerous factors may have contributed to the limited success of CCP in severe COVID-19, including recipient variables, such as the timing of CCP administration relative to the onset of symptoms, concomitant COVID-19 therapies, and underlying comorbidities. Additionally, donor CCP heterogeneity, including anti–SARS-CoV-2 antibody titers, variant matching, and virus neutralization titers, complicates the ascertainment of risks and benefits of CCP in specific populations. It is also possible that other plasma components beyond neutralizing antibodies may mediate antiviral or antiinflammatory effects (13).

While antigen binding titers are commonly assessed, non–neutralizing antibody functions of CCP mediated via Fcγ receptors (FcγRs) are less well characterized in clinical studies. These antibodies can drive antibody-dependent cellular cytotoxicity (ADCC), antibody-dependent cellular phagocytosis (ADCP), or antibody-dependent cytokine release. The CONCOR-1 trial (7) reported that higher SARS-CoV-2–specific neutralization and ADCC activity in donor CCP was associated with reduced intubation and mortality in CCP recipients even though CCP showed no overall effect. Within this same study, IgG binding against the full-transmembrane SARS-CoV-2 spike was associated with potential harm. Variable CCP titers from different suppliers also impacted results, suggesting that the specificity of CCP binding could have both positive and negative effects.

Preexisting immunity against seasonal coronaviruses (sCoVs) and antibody cross-reactivity among SARS-CoV-2 variants may influence CCP efficacy. In early 2020, cross-reactive antibody responses against SARS-CoV-2 spike in some pre-pandemic sera were found to neutralize SARS-CoV-2 infection in vitro (14, 15), likely because of memory responses to conserved sCoV epitopes. The impact of these cross-reactive responses on COVID-19 clinical outcomes is unclear. While one study has shown a beneficial effect (15), others have demonstrated the opposite, with higher antibody levels associated with severe COVID-19 (16–18). Better outcomes were observed in cancer patients receiving CCP with higher anti–OC43 and anti–HKU-1 spike IgG-specific antibodies (19).

In severe COVID-19, in the absence of CCP treatment, elevated and skewed immune responses are associated with poor clinical outcomes, including elevated proinflammatory cytokines (20–24), high anti-spike IgG titers (25–28), high titers of sCoV–cross-reactive antibodies (16, 17, 29–31), and high non–neutralizing antibody effector functions (32–36). In addition, during the early COVID-19 pandemic, longitudinal D-dimer trajectories were associated with disease severity and mortality (37, 38).

When evaluated as a homogeneous therapeutic product, CCP has not been found to associate with hazard when administered to more than 20,000 participants (10, 11, 39). In contrast, this study evaluated CCP on a donor level to assess whether unique donors provided antibodies that negatively impacted the outcomes of patients with severe COVID-19. The presence of “harmful” individual donors may contribute to the diminished effect of “beneficial” donors when CCP is evaluated as a homogeneous therapeutic in the hospital setting. In this retrospective study of 304 CCP units given to 414 patients hospitalized for severe COVID-19 within the Mount Sinai Health System during the first 4 months of the COVID-19 pandemic, we conducted an immunological analysis of CCP units given to recipients with adverse D-dimer trajectories after CCP administration. By studying the donor-recipient network, we found that poor outcomes appeared to cluster non-randomly around a small number of high-risk CCP units. Elevated SARS-CoV-2 functional antibody responses and increased cross-reactivity with seasonal OC43 coronavirus were associated with high-risk versus low-risk CCP.

## Results

### CCP recipient cohort.

**Figure 1:**
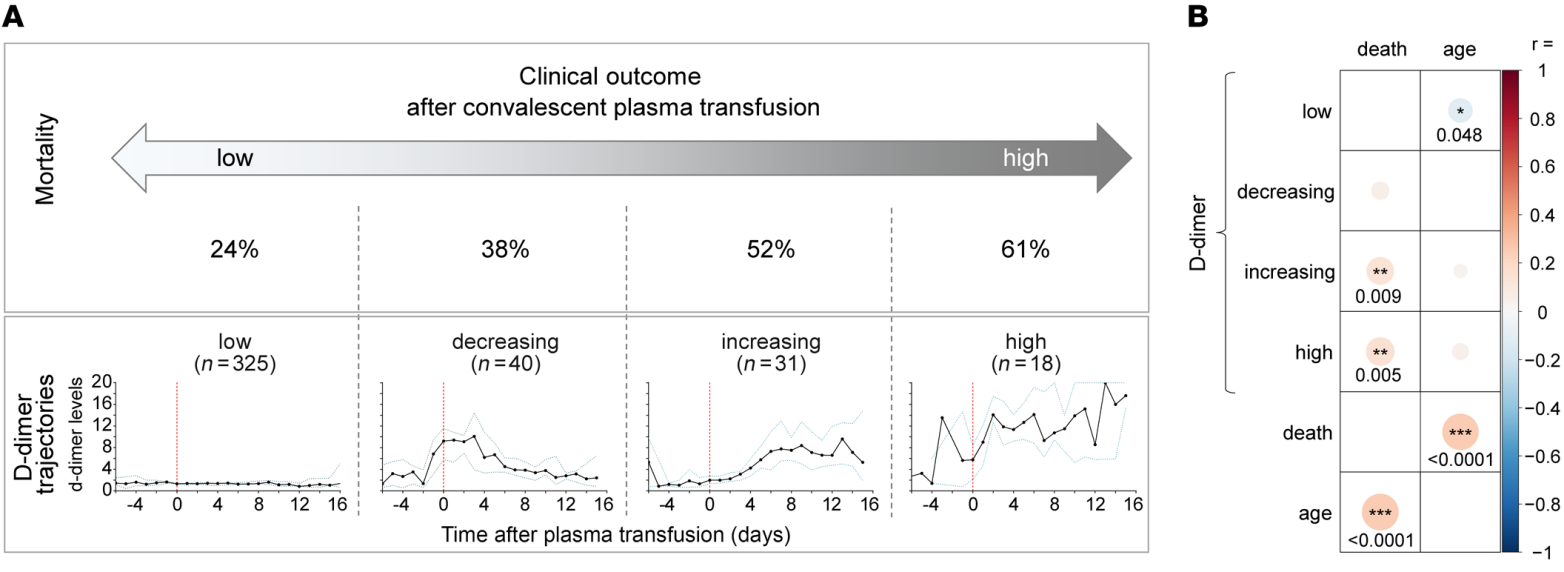
Clinical characteristics of severely COVID-19–infected patients after convalescent plasma transfusion. (**A**) D-dimer trajectories of COVID-19 patients (*n* = 414) and their association with mortality rates. Analysis of D-dimer levels over time revealed 4 distinct D-dimer trajectory groups using latent class modeling: low, decreasing, increasing, and high. Mean values and 95% confidence intervals (dotted lines) are shown over 15 days after plasma transfusion. (**B**) Correlation of death and age and with each D-dimer trajectory group. The correlogram is color-coded according to Spearman’s rank coefficient (*r*) between the respective pairwise variables. Asterisks indicate statistically significant correlations (Spearman’s rank, *P < 0.05, **P < 0.01, ***P < 0.001).

Within our CCP recipient cohort (*n* = 414) group, we characterized the D-dimer trajectories of patients following treatment with CCP to explore the potential connection between variable components in CCP that may induce or exacerbate coagulopathy and subsequent mortality. Using latent class modeling, 4 distinct groups were identified among CCP recipients concerning the D-dimer trend: those with persistently low levels (*n* = 325), those with decreasing levels (*n* = 40), those with increasing levels (*n* = 31), and those with persistently high levels (*n* = 18) after CCP receipt. Individual D-dimer trajectories of all patients (Supplemental Figure 1; supplemental material available online with this article; https://doi.org/10.1172/JCI181136DS1) and median D-dimer levels of each group (Figure 1A) are shown. We then examined mortality rates by patients’ D-dimer trajectories. Patients with either persistently low or decreasing D-dimer trajectories after CCP treatment had 24% and 38% mortality, respectively. Patients with either increasing or persistently high D-dimer trends after CCP administration experienced higher mortality rates of 52% and 61%, respectively (*P* < 0.0001, χ^2^ test). These trends gave rise to a hypothesis that some factor(s) associated with CCP donors can give rise to coagulopathy, driving high endpoint D-dimer trends in a subset of recipients.

The baseline characteristics of patients in the 4 D-dimer trajectory groups are shown in Table 1. Age, sex, race, and BMI did not significantly vary between the D-dimer groups (*P* = 0.2, *P* = 0.7, *P* = 0.98, *P* = 0.1, respectively). While mortality correlated significantly with age (*P* < 0.0001) and with high (*P* = 0.005) or increasing (*P* = 0.009) D-dimer trajectory, there was only a weak association with age and low D-dimer trajectory (*P* = 0.048), indicating that age and D-dimer trajectory are otherwise 2 independent correlates of clinical outcome (Figure 1B). The blood group was also not correlated with any D-dimer trajectory, age, or mortality (Supplemental Figure 2).

### Donor-recipient network connections with D-dimer.

**Figure 2:**
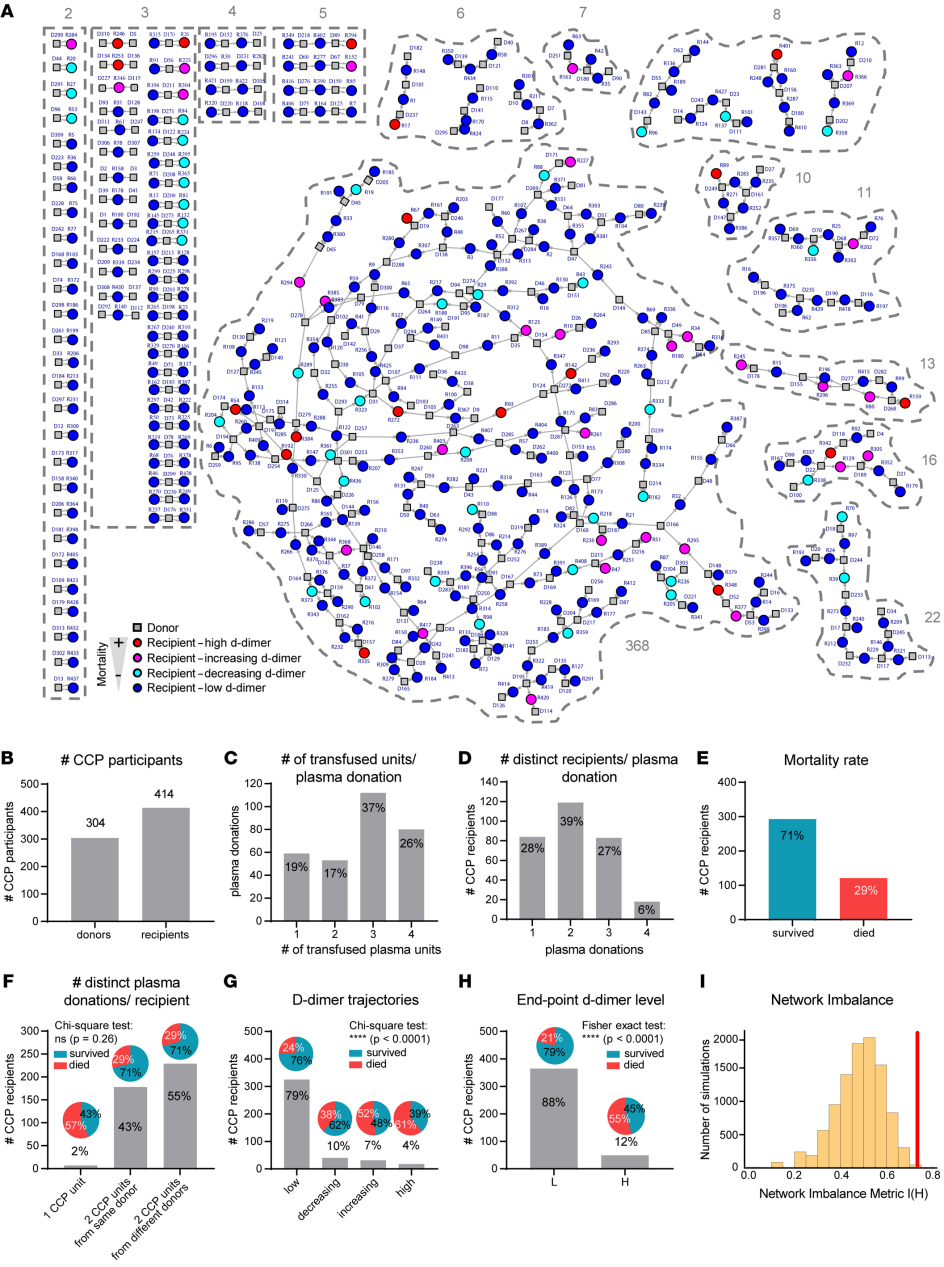
COVID convalescent plasma donor-recipient network analysis reveals non-random (clustered) distribution of H recipients. (**A**) Donors (squares) are connected to recipients (circles) in interconnected networks. Recipients are colored according to D-dimer trajectory, with high and increasing trajectories (red, magenta) and low and decreasing trajectories (blue, turquoise). The network was used to assign a D-dimer risk score for each unit of plasma. (**B**) Network of participants, donors, and recipients. (**C**) Total donations per donor. (**D**) Number of distinct recipients per donor. (**E**) Mortality rate in plasma recipients. (**F**) Number of distinct donors per recipient. (**G**) Mortality rate segregated by D-dimer risk score. (**H**) Mortality rate segregated by high versus low D-dimer risk score. (**I**) Permutation analysis of the network plots the probability of H individuals clustering in proximity to each other [I(H)] over 10,000 simulations compared with p(H) in the actual CCP cohort (red line). Differences in groups were measured by χ^2^ test (**F** and **G**) or Fisher’s exact test (**H**).

In the cohort of donors and recipients, we mapped the connections between each CCP and the recipients of that CCP. In the network diagram, each plasma transfusion is represented directionally by an arrow from donor to recipient, with each donor connected to multiple recipients. A total of 89 clusters were formed (Figure 2A). The majority of donors and recipients were interconnected in one large cluster (51%, *n* = 368, labeled as 368 in Figure 2A), 23% (*n* = 168) of all CCP participants were represented in small clusters of 2 or 3 subjects, and 25% (*n* = 182) of the CCP cohort was found in medium-sized clusters of 4–22 connecting subjects. Recipients with high or increasing D-dimer trajectories (red and magenta circles) associated with poor clinical outcomes were found in small, medium, and large clusters.

The donor-recipient network illustrated in Figure 2A includes 304 unique donors and 414 recipients (Figure 2B). Each plasma donation was administered to up to 4 recipients, with a majority of units given to 3 recipients (Figure 2C). The number of distinct recipients per donor plasma ranged between 1 and 4, with 2 individual recipients being most common (39%) (Figure 2D). Among the 414 recipients, mortality was 29% (Figure 2E). Most recipients were transfused with 2 CCP units (98%, *n* = 407), and 7 patients received a single unit. Recipients of 2 plasma units received them from either 1 or 2 distinct donors, with 43% of the individuals receiving CCP from a single donor (Figure 2F and Supplemental Figure 3, A–C). There was no significant difference in the mortality rate between recipients receiving their CCP units from the same or 2 different donors (*P* = 0.26, χ^2^ test). However, the mortality rate was higher in patients receiving only a single unit; 57% of these died, compared with 29% of patients receiving 2 units from the same or 2 different donors. The 2% of CCP recipients who received only a single infusion may have had transfusion reactions that precluded the administration of a second unit of CCP. However, the reasons for the higher mortality in this small subgroup are unclear. The distribution of D-dimer trajectories and their mortality rates in the CCP cohort is shown in Figure 2G and Supplemental Figure 3, D–F. Most recipients (79%) had persistently low D-dimer trajectories, with the rest developing decreasing or increasing D-dimer levels or maintaining high D-dimer levels after CCP transfusion. Overall, the mortality rate differed in these patient groups (*P* < 0.0001, χ^2^ test), with the highest in recipients with increasing and high D-dimer trajectories (52%, 61%). A significant difference was also found when endpoint D-dimer levels were examined, with low and decreasing D-dimer trajectories designated as low (L) and increasing and high D-dimer trajectories designated as high (H) (Figure 2H). Twenty-one percent of CCP recipients died in the group with low endpoint D-dimer levels compared with 55% in the group with high endpoint D-dimer levels (*P* < 0.0001) (Figure 2H).

To further examine the hypothesis that some plasma may be more closely associated with adverse D-dimer trajectory following infusion, we next performed a network analysis of the donors and recipients to examine the randomness of association between CCP recipients with high endpoint D-dimer levels and individual CCP donors within the network. Our analysis examined whether the distribution of poor outcomes tends to cluster around certain plasma donations. A CCP risk score was assigned based on the degree to which a unique donor’s plasma was connected to adverse D-dimer trajectories in the recipients of that plasma. Because these adverse D-dimer trajectories occurred with relatively low frequency, we could identify specific CCP donors that associated with increasing or persistently high D-dimer trends at a rate greater than would be randomly expected.

To statistically quantify whether an unfavorable clinical outcome of recipients (indicated by D-dimer trajectories) in this cohort was associated with specific “high-risk” donors, a permutation analysis of the network was performed. As a null hypothesis, we assumed that high and low D-dimer outcomes are independent of the donors. To test this hypothesis, we computationally created artificial versions of the network in which the observed high and low D-dimer outcomes were randomly distributed among the recipients of the network in Figure 2A. To achieve this, data were randomly resampled 10,000 times under the constraint that the number of low (L) and high (H) endpoint D-dimer levels among the recipients in each randomized resampling was the same as given in the original data. For each resampling, we studied the tendency of preferential clustering of H recipients in proximity to each other on the network. We defined a metric, which we called I(H), that measures the imbalance of clustering: if the likelihood of finding an H recipient in the neighborhood of another H recipient is the same as that of finding it in the neighborhood of an L recipient, then I(H) = 1/2. A deviation from ½ signals the presence of clustering of H recipients in the proximity of each other. The probability distribution of the imbalance metrics among the 10,000 resampled networks is shown by the yellow histogram in Figure 2I. Because of the random assignment of H and L labels in these simulations, the distribution of I(H) in this histogram is centered around 0.5, indicating that in the majority of simulations, H recipients were just as likely to occur near L recipients as they were near other H recipients. Thus, they were not associated with specific donors. In the observed network, however, the tendency of preferential clustering of H recipients in proximity was found to be I(H) = 0.729, shown in the red bar in Figure 2I. The resampling procedure implemented here allows for direct calculation of the *P* value (that is, the probability of the null hypothesis being true), yielding *P* ≈ 0.0007 (see Methods for details). Hence, in the observed network, H recipients were significantly more likely to be found together in clusters than in the randomized simulations. We thus conclude that H recipients in this donor-recipient network are non-randomly associated with distinct plasma donors, i.e., that adverse outcomes among recipients are driven at least in part by certain “high-risk” donors.

### Antibody binding profiles of CCP.

**Figure 3:**
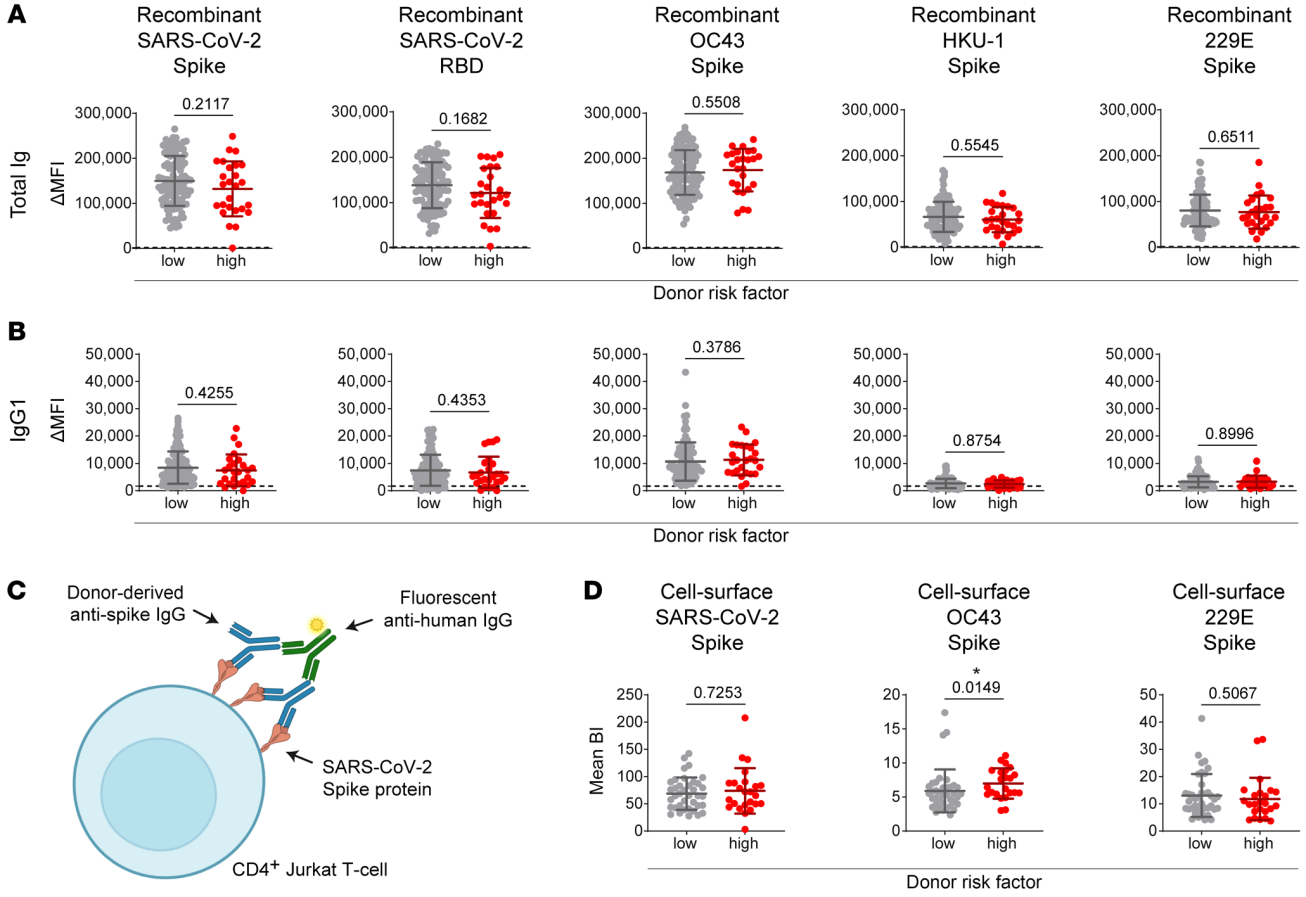
Higher cell-surface OC43 binding IgG titers are found in high-risk donor CCP. (**A** and **B**) Binding of total Ig (**A**) and IgG1 (**B**) from donor CCP to recombinant proteins of SARS-CoV-2 spike, SARS-CoV-2 RBD, OC43 spike, HKU-1 spike, and 229E spike using a Luminex bead-based multiplex assay comparing differences between antibodies associated with a high (D-dimer score < 1.25) versus low score (D-dimer score ≥ 1.25). Each experiment was measured in duplicate; the means with SD for a total of *n* = 135 CCP samples, including *n* = 109 low-risk and *n* = 26 high-risk samples, are shown. (**C**) Antibody binding assay using cell surface–expressed spike proteins. (**D**) Binding of purified donor CCP IgG against SARS-CoV-2, OC43, and 229E was measured using the assay shown in **C**. BI, binding index. The data represent *n* = 2 experiments measured in duplicate, showing means with SD for *n* = 61 CCP samples, including *n* = 38 low-risk and *n* = 24 high-risk samples. Differences in responses between “low-risk” and “high-risk” CCP donors were measured by Mann-Whitney tests (**A**, **B**, and **D**). Asterisk indicates statistical significance (*P < 0.05).

We hypothesized that CCP provided by high-risk donors — those whose plasma units were associated with increasing or high D-dimer trajectories in recipients — have unique antibody characteristics that may be responsible for D-dimer activation. Based on the network analysis, we then grouped all CCP donors into high- or low-risk plasma groups based on a D-dimer scoring system (Supplemental Figure 4), which takes the D-dimer trajectories of all neighboring recipients of each donor into account. To test our hypothesis, we measured antibody activities in a subset of CCP from high- and low-risk donors. We measured the total spike-binding Ig, IgG1, IgA1, and IgM using a multiplex bead-based assay. There were no significant differences in anti-spike antibody titers among CCP with low versus high D-dimer risk scores (Figure 3, A and B, and Supplemental Figure 5A). Previously, several studies have shown that the titers of antibodies targeting the receptor binding domain (RBD) region of the SARS-CoV-2 spike correlate with neutralizing activity (40–43). Therefore, we also quantified the level of anti-RBD total IgG, IgG1, IgM, and IgA and detected no significant differences between CCP with low and high risk scores (*P* = 0.17, *P* = 0.44, *P* = 0.21, *P* = 0.52) (Figure 3, A and B, and Supplemental Figure 5B). The overall high titers of CCP are consistent with the CCP donor protocol whereby CCP donors were prescreened for high anti-spike and anti-RBD levels.

Several studies examining the humoral immune correlates of COVID-19 severity have observed a positive correlation between betacoronavirus (β-CoV) cross-reactivity and disease severity (16, 17, 29–31). We next measured the titers of antibodies directed against seasonal coronaviruses (sCoVs) in CCP using recombinant spike proteins. We observed no significant differences in anti-spike antibody titers directed against OC43 (β-CoV), HKU-1 (β-CoV), or 229E (α-CoV) spike proteins in low- versus high-risk CCP (Figure 3A). However, we noticed overall higher Ig total and IgG1 titers for OC43 compared with SARS-CoV-2.

To assess IgG binding to native forms of spike expressed on the surface of cells, a cell-based viral spike (S) display system was used to quantify the levels of anti-S IgG binding to cell-associated, native-like forms of viral envelopes (31, 44) (Figure 3C). Purified IgG from each CCP was incubated with cells transduced with expression vectors producing β-CoV spike. We detected no significant differences in the levels of IgG against the spike protein of SARS-CoV-2 or 229E (*P* = 0.73, *P* = 0.51) (Figure 3D and Supplemental Table 1). However, significantly higher levels of anti-OC43 IgG in the CCP were associated with high versus low D-dimer scores (*P* = 0.01, adjusted *P* = 0.08) (Figure 3D and Supplemental Table 1). This finding indicates that anti–OC43 spike antibodies in donor CCP or differences in epitope targeting are associated with high endpoint D-dimer trajectories in CCP recipients.

We also quantified the levels of CCP IgG1 binding to the S1 and S2 subunits of the SARS-CoV-2 S protein and the level of anti-nucleocapsid (anti-N) IgG1. Still, we did not find significant differences between CCP in the high- and low-risk categories (*P* = 0.67, *P* = 0.20, *P* = 0.99) (Supplemental Figure 5B). Binding assays to recombinant protein immobilized on Luminex beads did not detect significant differences in the levels of cross-reactive antibodies, including anti-OC43 IgG. This also may denote that there are differences in probing of antibody binding with recombinant proteins versus native-like proteins expressed on the surface of cells.

### Functional antibody characterization of CCP.

**Figure 4:**
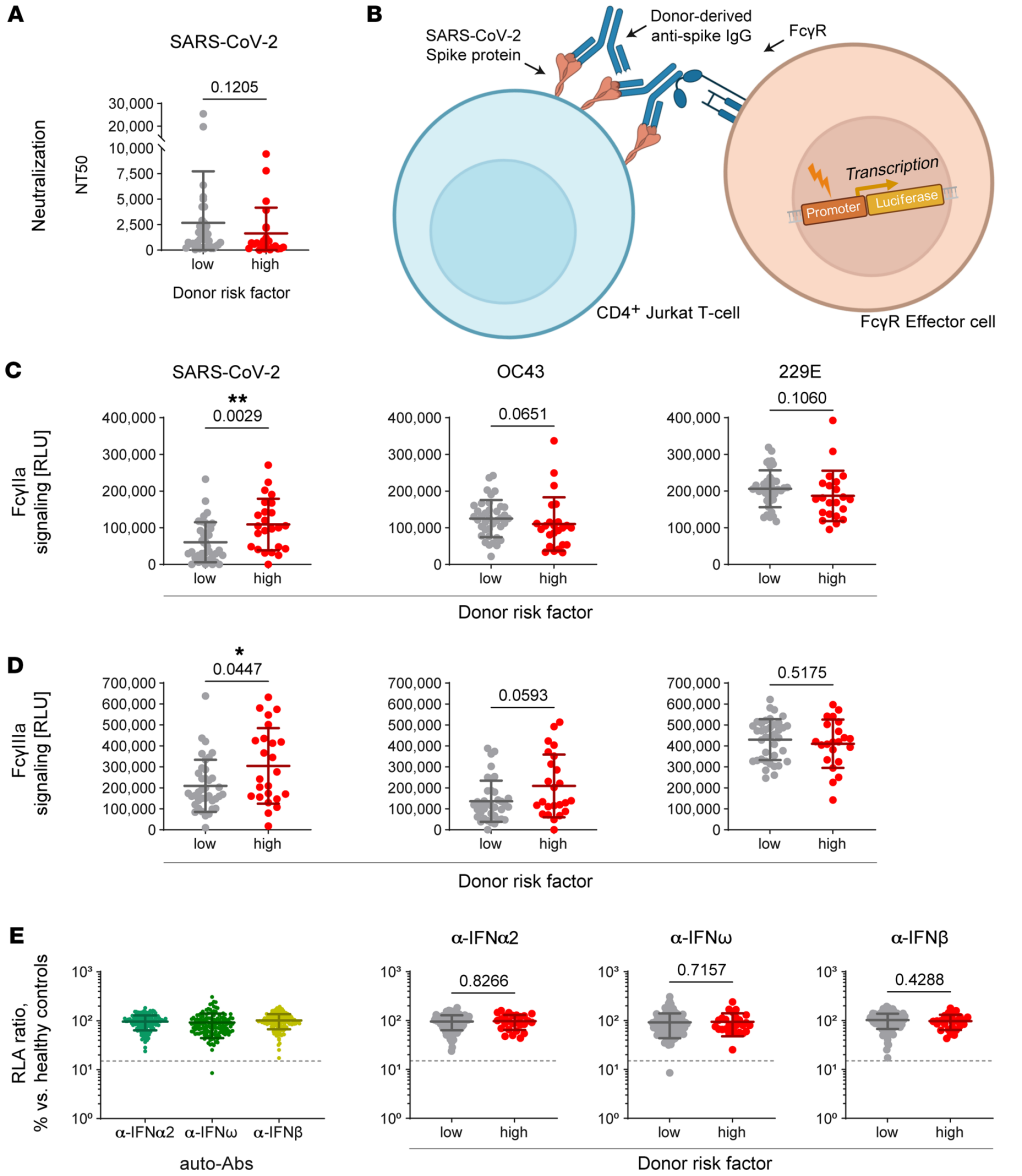
Antibody functions of low- and high-risk donor CCP. (**A**) Neutralization of CCP plasma against SARS-CoV-2 using SARS-CoV-2 spike pseudotyped particles incubated with plasma from SARS-CoV-2 convalescent plasma (CCP) donors, tested on 293T cells expressing ACE2 receptor. The experiment was measured in triplicate; the means with SD for a total of *n* = 61 CCP samples, including *n* = 38 low-risk and *n* = 23 high-risk samples, are shown. (**B**) The FcγR signaling assay measured the ability of purified IgG to bind to the surface of Jurkat cells expressing SARS-CoV-2 spike protein, which were then cocultured with FcγR signaling effector cells. (**C** and **D**) FcγRIIa (**C**) and FcγRIIIa signaling (**D**) of low- versus high-risk CCP donor IgG against SARS-CoV-2, OC43, and 229E using the assay shown in **B**. The experiment was performed in duplicate. Results are shown as the means with SD for a total of *n* = 62 CCP samples, including *n* = 38 low-risk and *n* = 24 high-risk samples. (**E**) The blocking activity of autoantibodies in donor CCP against IFN-α2, IFN-ω, and IFN-β was determined with a reporter luciferase cell line. Samples were considered neutralizing if luciferase induction was below 15% of the median values for healthy controls (dashed lines). The experiment was measured in triplicate; the means with SD for a total of *n* = 135 CCP samples, including *n* = 109 low-risk and *n* = 26 high-risk samples, are shown. Differences in responses between “low-risk” and “high-risk” CCP donors were measured by Mann-Whitney tests (**A** and **C**–**E**). Asterisks indicate statistical significance (*P < 0.05, **P < 0.01).

We next measured the neutralization activity of CCP against SARS-CoV-2 using a pseudoparticle neutralization assay (Figure 4A). As for the SARS-CoV-2 antibody binding titers, we observed no significant differences in the anti–SARS-CoV-2 neutralization activity among CCP in high versus low risk categories (*P* = 0.12). In addition to neutralizing activity, polyclonal antibody responses were further characterized by their antigen and epitope targeting profiles, Fc-mediated effector functions, and immunomodulatory properties, all collectively contributing to inflammation and clearance of pathogens and infected host cells.

Interaction with FcγRs on the surface of innate immune cells or platelets can activate cell clearance, inflammation, or clotting. We measured the ability of cell-based forms of the spike antigen to activate 2 of the primary FcγRs that initiate proinflammatory immune responses: FcγRIIa and FcγRIIIa (Figure 4B). Interestingly, we observed that CCP IgG from the high-risk group activated significantly higher levels of FcγRIIa against the SARS-CoV-2 spike protein, as compared with CCP from the low-risk-score group (*P* = 0.003, adjusted *P* = 0.03) (Figure 4C and Supplemental Table 1). We also detected significantly higher SARS-CoV-2–specific FcγRIIIa signaling from CCP IgG in the high- versus low-risk-score group (*P* = 0.04, adjusted *P* = 0.14). Since we detected higher titers of anti–OC43 spike IgG in CCP associated with high risk scores, we examined the levels of FcγRIIa and FcγRIIIa activation against the spike proteins of OC43 and 229E. In comparing high- versus low-risk-score donors, we observed a non-significant trend toward higher levels of FcγRIIa and FcγRIIIa signaling against the spike protein of β-CoV OC43 (*P* = 0.07, *P* = 0.06; adjusted *P* = 0.14, *P* = 0.14) (Figure 4, C and D, and Supplemental Table 1). This implies that the anti-OC43 antibodies that change the overall epitope binding profiles, or epitope targeting ratios, may also add to higher levels of FcγR signaling observed in these CCPs against SARS-CoV-2 spike.

Beyond cross-reactivity and enhanced non-neutralizing Fc-mediated antibody signatures, previous studies have observed that COVID-19 severity positively correlated with the levels of anti-IFN autoantibodies (8). To examine whether autoimmune humoral signatures were present in the CCP given to recipients with high D-dimer trajectories, we quantified the levels of autoantibodies against IFN-α2, -ω, and -β. Only one CCP of the 139 examined possessed autoantibodies targeting IFN-ω (Figure 4E). This donor CCP was associated with a low-risk D-dimer score.

## Discussion

Some COVID-19 treatment studies initially revealed that CCP recipients had better outcomes with high-titer CCP than with low-titer CCP, suggesting that IgG titer could impact disease progression or severity of illness (1, 3). Moreover, the efficacy of monoclonal antibodies in early disease further suggested that antibodies can prevent severe disease. However, the lack of demonstrable efficacy of CCP in large RCTs raises the question of whether some antibody responses can have adverse effects when the antibodies are administered during severe COVID-19. Hyperimmune globulin, which contains the purified IgG from CCP, is safe in healthy individuals (45), though, like CCP, it has shown no benefit in the treatment of severe COVID-19. In patients with preexisting antibody titers, those receiving hyperimmune globulin had a higher risk of death or serious adverse events compared with placebo controls (46). This led us to hypothesize that in severe disease, some antibodies may be deleterious and, more specifically, may be associated with coagulopathy shortly after administration.

In this study, we connected data from an interconnected network of donors and recipients to assign a D-dimer risk score to each plasma unit based on its frequency of association with H D-dimer trajectories. This revealed that H plasma was non-randomly associated with higher D-dimer risk scores. This pattern would not be expected if plasma only had a positive or neutral impact on the D-dimer trajectory. This warranted a closer look at the functional properties of antibodies in the plasma associated with H D-dimer trajectories. Notably, while H D-dimer trajectories correlated with increased mortality, mortality did not cluster significantly around specific plasma units, suggesting that some deaths were not associated with D-dimer risk, weakening the association with specific plasma units.

Considering that anti–CoV-2 S titers in CCP administered to patients can vary, we note that the CCP donors in this study were prescreened for high SARS-CoV-2 antibody titers using an ELISA assay developed at Mount Sinai Hospital (47). In our analysis, we measured anti–SARS-CoV-2 spike antibody titers using bead-based (Luminex) and cell-based detection assays. Notably, SARS-CoV-2–specific antibody titers or the neutralization activity did not differ between H CCP and L CCP. Previous studies linked high SARS-CoV-2–specific antibody titers in CCP with positive clinical outcomes in recipients (1, 3, 48, 49). Yet here, lower titer was not associated with H D-dimer trajectory. Furthermore, we found no differences in neutralization activity among CCP samples related to D-dimer trajectories, suggesting that low neutralizing antibody titers are not associated with coagulopathy.

In this study, we observed elevated titers of potentially SARS-CoV-2–cross-reactive anti–OC43 spike antibodies in CCP administered to recipients with high endpoint D-dimer levels. This indicates a connection between high cross-reactive antibody titers in CCP and coagulopathy in recipients. Other studies examining the initial SARS-CoV-2 antibody responses during acute COVID-19 infection have found that disease severity was associated with elevated sCoV antibody titers (17, 29, 31, 50). A longitudinal study examining pre- and postinfection sCoV antibody levels found that higher preinfection sCoV antibody titers correlated with more severe COVID-19 (17). Another study linked antibody levels targeting β-CoV–conserved epitopes to fatal COVID-19 cases (16). Our findings revealed an increased ratio of anti-OC43 versus anti–SARS-CoV-2 antibody titers in high-risk CCP, suggesting that infusion of specific SARS-CoV-2 antibody targeting particular β-CoV–conserved epitopes is associated with adverse D-dimer trajectories.

In studies examining the correlates of severe COVID-19 there are indications that distinct functional antibody properties can distinguish between protective and detrimental inflammatory responses. Despite the robust correlation between high β-CoV antibody titers and disease severity, other studies have indicated that higher sCoV antibody titers correlate with protection and milder COVID-19 (15, 51–53). Garrido et al. observed that higher antibody titers targeting specific OC43–cross-reactive epitopes within the S2 subunit were associated with disease severity, while antibody targeting the HR2 fusion peptide region was associated with milder disease (31). This study indicates that the administration of convalescent plasma with certain specificities may modulate its therapeutic versus proinflammatory effects.

The observed differences in IgG binding profiles between the H and L D-dimer trajectory groups were detected with cell membrane–expressed coronavirus spike proteins. These proteins may provide a more native conformation of viral antigens and present different epitopes compared with the recombinant protein used in the Luminex assays (44, 54). These differences underscore the importance of identifying specific functional antigens that represent the forms of antigen that are exposed during infection.

Beyond elevated anti-OC43 antibody titers, we observed that FcγRIIa activation and, to a lesser extent, FcγRIIIa activation against the SARS-CoV-2 spike correlated with high D-dimer and higher mortality rates in CCP recipients. While FcγRIIa can potentially induce ADCC in monocytes, it also induces cellular activation, ADCP, and cytokine expression from several innate immune cells, such as monocytes, macrophages, neutrophils, and platelets. The results here support a model whereby immune complexes in patients with viremia and specific IgG may induce FcR-mediated hyperinflammatory coagulopathy responses.

Severe COVID-19 often includes severe pulmonary inflammation, vasculature damage, and increased thrombotic complications (55–58). Hospitalized patients often display coagulation abnormalities accompanied by elevated fibrinogen, D-dimer, and thrombocytopenia (59, 60). D-dimers are the principal breakdown fragment of fibrin and act as a surrogate biomarker for thrombosis (localized coagulation) and fibrinolysis (61). By associating D-dimer trajectory with high-risk CCP, we implicate a model whereby immune complexes may drive thrombosis and fibrinolysis that contributed to coagulopathy-related mortality in CCP recipients.

Platelets, key initiators of thrombosis, can be activated by immune complexes through FcγRIIa, releasing inflammatory mediators such as C5a in response to infectious agents. FcγRIIa is the only FcγR expressed on platelets. Notably, several studies have consistently observed higher levels of platelet activation in severe COVID-19 cases (35, 62–64). Consistent with our findings, Apostolidis et al. showed that antibodies from patients with severe COVID-19 induced higher levels of platelet activation via FcγRIIa signaling than antibodies from non-hospitalized COVID-19 convalescent or healthy controls (35).

We acknowledge several limitations in this study. Firstly, because we studied hospitalized COVID-19 patients during the first wave of the pandemic in a SARS-CoV-2 antibody–naive population, the results may not apply to current populations. Additionally, while the study is, to our knowledge, unique in its ability to cross-compare outcomes in individuals who received the same unit of plasma within the donor-recipient network, this was a focused study on a single site, and of a limited sample size. The sample size was sufficient to achieve statistical power for the demographic and network analysis. However, the assessment of antibody features in donor plasma was also constrained by the number of available donor CCP samples. Besides the clear differences between H and L CCP donors described earlier, additional trends were observed between the 2 groups (e.g., for OC43 FcγRIIIa), which may be statistically significant but lacked sufficient power to be conclusively determined. However, even if these trends prove to be significant, they would not contradict the findings presented here and would not alter the overall conclusions.

Despite these limitations, the unique non-random distribution of high-risk plasma in an interconnected CCP donor-recipient network and the similarity of the antibody profiles associated with D-dimer trajectory to severe disease-related profiles here provide a strong rationale to further explore mechanisms of immune complex–mediated coagulopathy as a major driver of CoV-2 pathology.

## Methods

### Sex as a biological variable.

Male and female participants were enrolled in this study, which was open to all sexes. Sex did not significantly vary between the different D-dimer groups (*P* = 0.7).

### Eligibility and selection of convalescent plasma donors and recipients.

CCP donors were prescreened for SARS-CoV-2 antibody titers and referred to a plasma donation center as previously described (2). During the period of this study, CCP recipients were treated under 2 separate Food and Drug Administration (FDA) emergency use authorization pathways, initially by single-patient emergency investigational new drug (eIND) applications to the FDA (March 24 through April 9, 2020) and then under the Expanded Access Program (EAP) administered by the Mayo Clinic (April 10 through August 29, 2020) (65). CCP recipients were all treated between March 28, 2020, and June 28, 2020. The eligibility criteria for CCP administration under single-patient eIND authorization and through the EAP have been previously described (2, 39, 66). Most CCP recipients were transfused with 2 units of ABO-type-compatible CCP. Of the 414 CCP recipients, 43% (*n* = 178) received both units from a single donor, 55% (*n* = 229) received units from 2 different donors, and 2% (*n* = 7) received only a single unit.

### Follow-up of patients receiving convalescent plasma transfusion.

Plasma recipients’ records were reviewed for longitudinal D-dimer for the hospital stay with up to 15 days after plasma therapy. These involved 414 plasma recipients. Baseline data were collected, including age, sex, ethnicity, obesity, weight loss, hypertension, acute respiratory distress syndrome, chronic obstructive pulmonary disease, asthma, pulmonary circulation disease, sleep apnea, chronic kidney disease, end-stage renal disease, renal failure, liver disease, chronic viral hepatitis, coronary artery disease, atrial fibrillation, heart failure, valvular disease, peripheral vascular disease, chronic blood loss anemia, coagulopathy, fluid and electrolyte disorders, cancer, HIV, hypothyroidism, rheumatoid arthritis, cerebral infraction, paralysis, psychoses, and depression; as well as the use of antibiotics, steroids, antiplatelet therapy, tocilizumab, remdesivir, hydroxychloroquine, or azithromycin, intubation, and tracheostomy status during the hospital stay. Day 0 for convalescent plasma recipients was defined as the day on which they received plasma transfusion. Plasma units were received from a total of 304 plasma donors, who donated blood up to 4 times.

### Trajectory analysis.

We examined D-dimer trajectories to investigate their association with clinical progression and an acquired prothrombotic state over time. Trajectory analyses provided the opportunity to perform subgroup analyses of patients based on particular pathological patterns. Trajectories were identified using an SAS macro named PROC TRAJ, which applies a multinomial modeling strategy to identify relatively homogeneous clusters of developmental trajectories within a sample population. Trajectory parameters were derived by latent class analysis using maximum likelihood estimation. In particular, the distinctive trajectories of D-dimer were derived by modeling of D-dimer as a function of the days within the 2 weeks following plasma transfusion. Distinct time points were created for each follow-up visit observed. The number of trajectories and degree of curvature were determined using the guidelines suggested by Jones et al. (65). Four trajectories were identified with quadratic, linear, quadratic, and quadratic curves corresponding to groups of increasing, persistently low, decreasing, and persistently elevated D-dimer levels, respectively. The output of PROC TRAJ includes the assignment of each patient to 1 of the trajectory groups. These group assignments were then analyzed using analysis of variance to identify differences between trajectory groups.

### Network clustering analysis.

To determine whether the clinical outcome of the recipients in this cohort was non-randomly distributed across donors, we adopted the methodology developed in Law et al. (67). All recipients were classified into 2 groups, based on their endpoint D-dimer levels: group L for those with low and decreasing D-dimer trajectories and group H for increasing and high D-dimer trajectories. Then, the imbalance metric, I(H), was calculated, which compares the prevalence of H recipients in the neighborhood of other H recipients versus the prevalence of H recipients in the neighborhood of L recipients. This requires a mathematical definition of a neighborhood in the context of the network in Figure 2A, which is provided in the Supplemental Analysis. This definition differs from some of the more standard metrics because it accounts for the fact that the relevant network consists of both recipients and donors, and it is the connections of donors with recipients that are central to this study. The number of H individuals is counted in the neighborhoods of H and L individuals and normalized; the formula for I(H) is presented in the Supplemental Analysis. In a situation in which the recipient status is completely independent of the donors, this metric would be equal to ½. An imbalance metric that is greater than ½ points to the existence of clustering of H individuals. To assess whether or not the deviation is statistically significant, we used simulations to create synthetic recipient-donor networks with random L/H assignment, but keeping the same numbers of H and L individuals and the same network structure as in Figure 2A. Then the probability distribution for the I(H) metric was obtained, and the *P* value calculated numerically. For a detailed description of the clustering analysis, please see the Supplemental Analysis.

### Calculation of the mean D-dimer score for donors as risk factor assessment.

We calculated a mean D-dimer score from the donor-recipient network to assess the “risk” associated with plasma from each of 304 CCP donors. Each CCP recipient was assigned a score of either 1 (if the D-dimer trajectory was persistently low or decreasing) or 2 (if the D-dimer trajectory was increasing or persistently high). Each donor’s mean D-dimer network score was calculated by the sum of scores from each directly connected recipient divided by the number of directly connected recipients. An example of the calculation is schematically shown in Supplemental Figure 4. Donors with a mean D-dimer score of 1–1.25 were defined as “low risk,” and donors with a score above 1.25 (up to 2) as “high risk.”

### Selection of plasma units for measuring antibody characteristics.

Multiplex bead antibody binding and anti-cytokine antibody reactivity were measured from all donors with available plasma segments (*n* = 135, 44% of cohort). A subset of samples (*n* = 61) was interrogated for cell-surface binding, neutralization, and Fc signaling. These assays were performed on all available plasma samples from donors with a mean D-dimer score above 1.25 (the “high-risk” donor group) (*n* = 24), as well as a random selection of plasma samples from donors with a mean D-dimer score of 1–1.25 (*n* = 37) to represent the “low-risk” donor group.

### Multiplex bead antibody binding assay.

Recombinant SARS-CoV-2 spike (full-length external region, amino acids 1–1213), SARS-CoV-2 RBD (amino acids 319–541), OC43 spike, HKU-1 spike, and 229E spike proteins were produced as described before (68). S1 (amino acids 16–685), S2 (amino acids 686–1213), and nucleoprotein (amino acids 1–419) antigens from SARS-CoV-2 were purchased from ProSci Inc. (97-087, 97-079, and 97-085). All SARS-CoV-2 antigens were derived from the Wuhan-Hu-1 (WA1) strain. Antigens were covalently coupled to magnetic beads using a 2-step carbodiimide reaction with the xMAP Antibody Coupling (AbC) Kit according to the manufacturer’s instructions (Luminex) as previously described (69, 70). Carboxylated xMAP beads were coupled to 2 μg protein per million beads for all coronavirus proteins or 4 μg protein per million beads for BSA, used as a negative control. The coupled beads were counted, diluted to a concentration of 500,000 beads/mL, and stored at 4°C for up to 1 month before use. Experiments were performed as previously described (72, 73). All samples were tested at a 1:200 plasma dilution and measured in duplicate, and results are shown as mean ﬂuorescence intensity (MFI).

### IgG purification.

Polyclonal IgG was isolated from plasma using a protein A/G spin column kit (Thermo Scientific, catalog 89950), followed by desalting using Zeba spin columns according to the manufacturer’s instructions (Thermo Fisher Scientific, 89892). IgG yields were quantified using an Easy-Titer IgG assay kit (Thermo Fisher Scientific, 23310).

### Anti–spike protein IgG determination using a cell-based assay.

The cell-based assay was performed as previously described (31). To quantify the levels of IgG binding to various coronavirus spike proteins, 293T cells (ATTC, CRL-3216) were transfected with SARS-CoV-2 (Sino Biological, VG40589-CF) (GenBank YP_009724390.1), OC43 (Sino Biological, VG40607-CF) (GenBank AVR40344.1), or 229E (Sino Biological, VG40605-CF) (GenBank APT69883.1) spike protein expression vectors. For this assay, 1 × 10^5^ 293T cells were plated in 10 cm plates and incubated at 37°C overnight. The next day, according to the manufacturer’s instructions, 4 mg of coronavirus spike expression vectors were transfected into 293T cells using PolyJet transfection reagent (SignaGen, SL100688). After 48 hours, 1 × 10^5^ 293T cells were plated per well into round-bottom 96-well plates. Cells were washed and incubated with 10 mg/mL of convalescent donor–derived IgG or negative control donor IgG and set at 4°C for 45 minutes. After primary antibody incubation, IgG opsonized cells were washed and incubated with 3 mg/mL of an APC-conjugated anti-human total IgG secondary antibody (Invitrogen, A21445) at 4°C for 25 minutes. Cells were then rewashed with PBS, and LIVE/DEAD Fixable Violet Stain (Invitrogen, L34964A) was used to stain cells for 10 minutes in the dark at room temperature. Lastly, cells were washed twice, fixed with 1.0% paraformaldehyde in PBS, and analyzed by flow cytometry (Invitrogen Attune NxT) (Supplemental Figure 6). The data were quantified using FlowJo software (Tree Star Inc.). The IgG-binding index was calculated by multiplication of the percentage of anti-spike IgG–positive cells by the APC signal’s median fluorescence intensity (MFI), as normalized to the average MFI of negative control IgG. To ensure that the relative differences between patient-derived IgGs were maintained, all IgGs were tested in parallel on the same day for each replicate.

### Neutralization assay.

Neutralization of SARS-CoV-2 was measured as previously described using the VSVΔG-rLuc SARS-CoV-2 spike pseudotyped particle system (codon-optimized Wuhan-Hu-1 isolate) (70). 293T-hACE2-TMPRSS2 (clone F8-2) cells (provided by the laboratory of Benhur Lee, Department of Microbiology, Icahn School of Medicine at Mount Sinai, New York, New York, USA) were seeded at a density of 3.5 × 10^4^ cells per well in a 96-well collagen-coated plate (Thermo Fisher Scientific, 152038) 24 hours before use in viral neutralization assays. All tested patient plasma samples were heat-inactivated (56°C for 30 minutes) before neutralizing studies. SARS-CoV-2 pseudotyped particles (COV2pp) were preincubated with 4-fold serially diluted plasma samples (1:10 to 1:40,960) in DMEM supplemented with 10% FBS for 30 minutes at room temperature before the COV2pp-sample mix was transferred to the seeded target cells. Infection was measured after 18–22 hours by luciferase activity. For this, infected cells were washed with Dulbecco’s PBS, lysed with passive lysis buffer, and processed for detection of *Renilla* luciferase (Renilla-Glo Luciferase Assay, Promega, E2720) in black-walled 96-well plates (Greiner, 655096). A Cytation3 apparatus (BioTek) using Gen5 software was used to read luminescence. The percentage of neutralization was calculated as follows:

 (Equation 1)
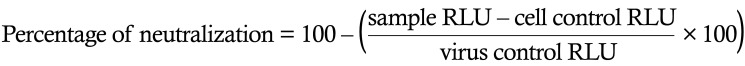


where RLU indicates relative light units. Fifty percent neutralizing titer (NT_50_) of SARS-CoV-2 was calculated as the reciprocal sample dilution, achieving 50% neutralization. Each sample and dilution was measured in triplicate. Three pre-pandemic plasma samples were used as a negative control.

### Fcγ receptor signaling assay.

FcγRIIa and FcγRIIIa signaling was assessed using a reporter cell coculture system as previously described (31). Briefly, 293T cells were transfected with the same spike expression vectors used for the cell-based assay and cocultured with either an FcγRIIa or an FcγRIIIa, CD4^+^ Jurkat reporter cell line (Promega, G7010), which expresses firefly luciferase upon FcγR activation. For this assay, SARS-CoV-2 spike–expressing 293T cells were plated in each well of a 96-well round-bottom plate. The cells were then preincubated with convalescent donor–derived IgG at 25, 5, or 0 mg/mL concentration. IgG opsonized 293T cells were then cocultured with FcγRIIa or FcγRIIIa reporter cells at a 2:1 reporter/target cell ratio for 24 hours at 37°C. After 24 hours, all cells were lysed with cell lysis buffer (Promega, E1531), and the levels of firefly luciferase activity were determined using a luciferase assay kit according to the manufacturer’s instructions (Promega, E1500). To quantify background (i.e., IgG activation–independent) luciferase production, reporter cells were cocultured with the spike-expressing 293T cells without any IgG. Background levels were subsequently subtracted from the signal to yield IgG-specific activation in relative light units. Luminescence was measured on a Cytation3 image reader using Gen5 software.

### Detection of anti-cytokine autoantibodies.

Autoantibody positivity was assessed for plasma samples as previously described by (8). Briefly, HEK293T cells were transfected with a plasmid containing the firefly luciferase gene under the control of the human ISRE promoter in the pGL4.45 backbone, and a plasmid constitutively expressing *Renilla* luciferase for normalization (pRLSV40). Cells were transfected with the X-tremeGene9 transfection reagent (Sigma-Aldrich, 6365779001) for 24 hours. Cells in DMEM (Thermo Fisher Scientific) supplemented with 2% fetal calf serum and 10% healthy control or patient serum (after inactivation at 56°C for 20 minutes) were either left unstimulated or stimulated with IFN-α2 (Miltenyi Biotec, 130-108-984) or IFN-ω (Merck, SRP3061) at 10 ng/mL or 100 pg/mL or IFN-β (Miltenyi Biotec, 130-107-888) at 10 ng/mL, for 16 hours at 37°C. Each sample was tested once for each cytokine and dose. According to the manufacturer’s protocol, cells were lysed and luciferase levels were measured with the Dual-Luciferase Reporter 1000 assay system (Promega, E1980). Luminescence intensity was measured with a VICTORX Multilabel Plate Reader (PerkinElmer Life Sciences). Activity values from firefly luciferase were normalized against the activity values from *Renilla* luciferase. These values were normalized against the median induction level for non-neutralizing samples and expressed as percentage. Samples were considered neutralizing if luciferase induction, normalized against *Renilla* luciferase activity, was below 15% of the median values for controls tested the same day.

### Software scripts and visualization.

Network analysis was done in program R, v4.1.21, using igraph and ggraph packages (71). Networks were created in directed mode, matching CCP donor and recipient pairs with superimposed metadata. Correlograms were generated using corrplot and RColorBrewer packages in R.

### Statistics.

Unless otherwise noted, data analysis was performed in Microsoft Excel 2013, GraphPad Prism 7.03, and R x64 version 4.1.21. Every dataset was tested for statistical normality (D’Agostino and Pearson), and this information was used to apply the appropriate (parametric or nonparametric) statistical test. The multiparameter pairwise correlation analysis for clinical and demographic variables used Spearman’s correlation. Correlation coefficients *r* and *P* values were calculated in GraphPad Prism. Multiplicity adjustments for *P* values were performed by the Benjamini-Hochberg method using the data.table and tidyverse packages in R (71, 72). Detailed statistical analysis for the network analysis is described in the Supplemental Analysis. In a post hoc power analysis, the comparison of a total of 365 CCP recipients with low endpoint D-dimer levels (88%) with 49 CCP recipients with high endpoint D-dimer levels (12%) achieved 82.1% power, in detecting a 22% difference in mortality in a 2-tailed Fisher’s exact test with a type I error of 5% (G*Power v3.1.9.4) (73). Differences in responses between “low-risk” and “high-risk” CCP donors were measured by Mann-Whitney tests. A post hoc power analysis comparing the SARS-CoV-2 neutralization of a total of 61 CCP donors with 37 “low-risk” and 24 “high-risk” donors showed 80% power in detecting a difference in means of 3,530 versus 1,600 with a standard deviation of 2,500 in a 2-tailed Mann-Whitney test with a type I error of 5% and the assumption that the data are normally distributed (G*Power v3.1.9.4).

Significance values are indicated as *P < 0.05, **P < 0.01, ***P < 0.001, ****P < 0.0001. Statistical tests were 2-tailed, and *P* less than 0.05 was considered significant.

### Study approval.

Convalescent plasma treatment and subsequent data analyses were performed with the oversight of the Icahn School of Medicine at Mount Sinai Institutional Review Board (IRB nos. 20-03393, 20-03574, and 20-03759). All CCP-treated patients or their legally authorized representatives gave informed consent for CCP treatment as an investigational therapy. As a retrospective analysis of compassionate-use treatment data, the study was neither prospectively designed nor registered on ClinicalTrials.gov, nor was a data safety monitoring board prospectively convened to oversee this study.

### Data availability.

Data are available upon request. Supporting data values associated with the main article and supplemental material are included in the Supporting Data Values file.

## Author contributions

SW, JAA, STHL, RAA, and BKC designed the study. THS contributed conceptual ideas. AW, SAA, JAA, NMB, and STHL designed the cohort. IB, AW, SAA, and STHL collected clinical data and biospecimens, which were processed by SW, EA, PC, and RAA. SW, EA, and RAA (laboratory of BKC) and AG and PB (laboratory of JLC) performed the experiments. SW, HML, and RAA processed and managed data. SW, HML, NLK, DW, and RD performed and/or interpreted the statistical analyses. SW, RAA, and BKC drafted the initial manuscript, with input from NLK, RD, THS, JAA, NMB, and STHL. All authors edited, reviewed, and approved the final manuscript.

## Supplementary Material

Supplemental data

Supporting data values

## Figures and Tables

**Table 1:**
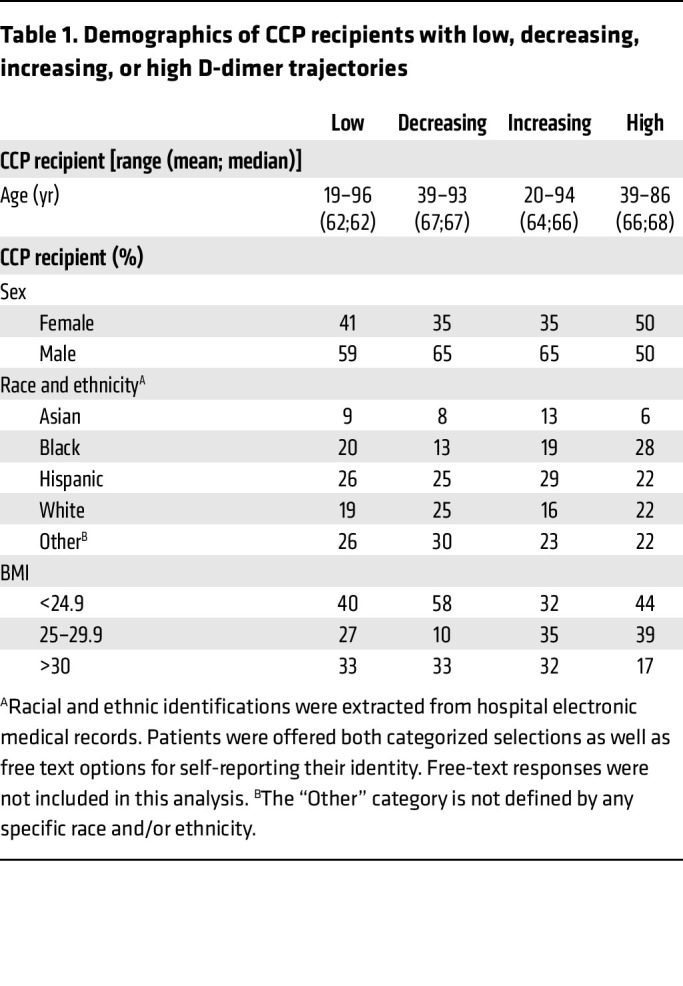
Demographics of CCP recipients with low, decreasing, increasing, or high D-dimer trajectories

## Version 1 03/17/2025

Electronic publication
