# Supplementary material for: Post-transfusion activation of coagulation pathways during severe COVID-19 correlates with COVID-19 convalescent plasma antibody profiles
Source: J Clin Invest. 2025 Mar 17;135(6):e181136. doi: 10.1172/JCI181136 (PMC11910229; doi:10.1172/JCI181136)
Supplement: Supplemental data [file jci-135-181136-s265.pdf]

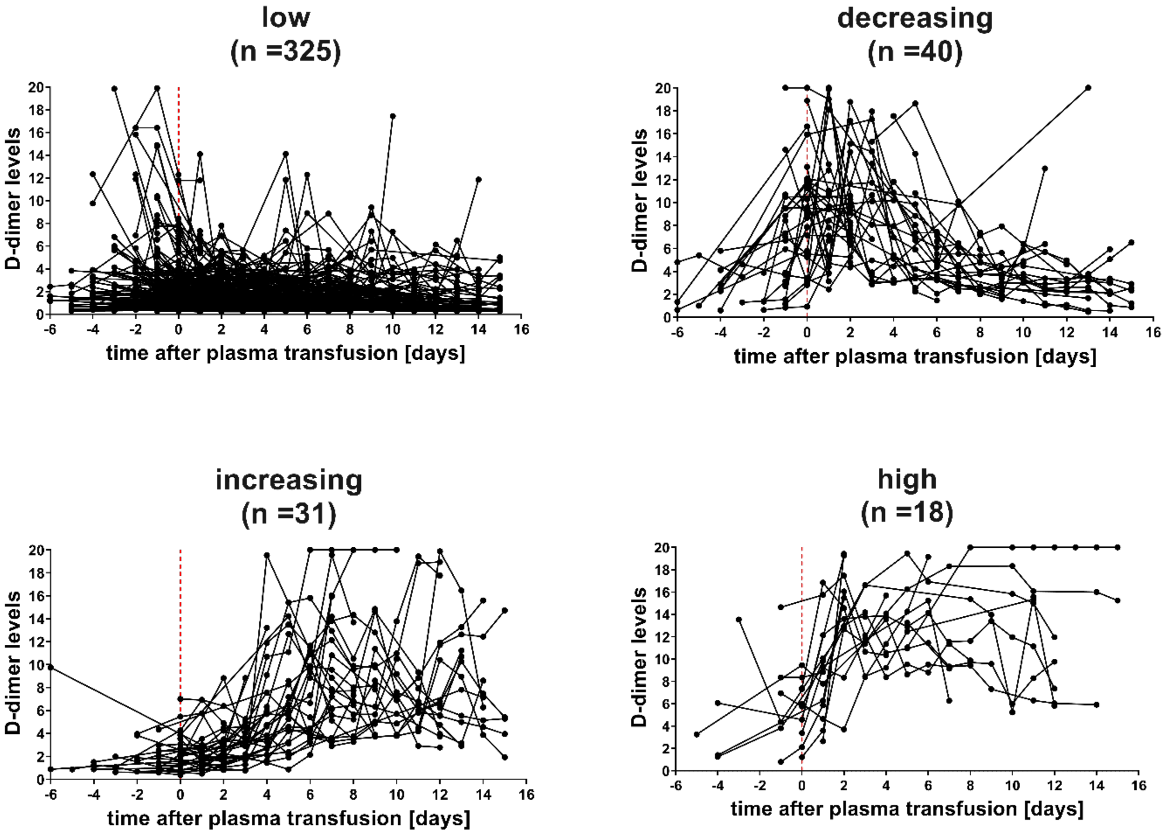

Supplemental Figure 1. Individual D-dimer trajectories of all CCP recipients.
Using latent class modeling, four distinct groups were identified amongst CCP recipients with respect to the D-dimer trend: those with persistently low levels (n= 325), those with decreasing levels (n = 40), those with increasing levels (n = 31) and those with persistently high levels (n = 18) after CCP receipt. Individual D-dimer trajectories of all patients are shown for a time frame of 6 days prior and 15 days post CCP transfusion.

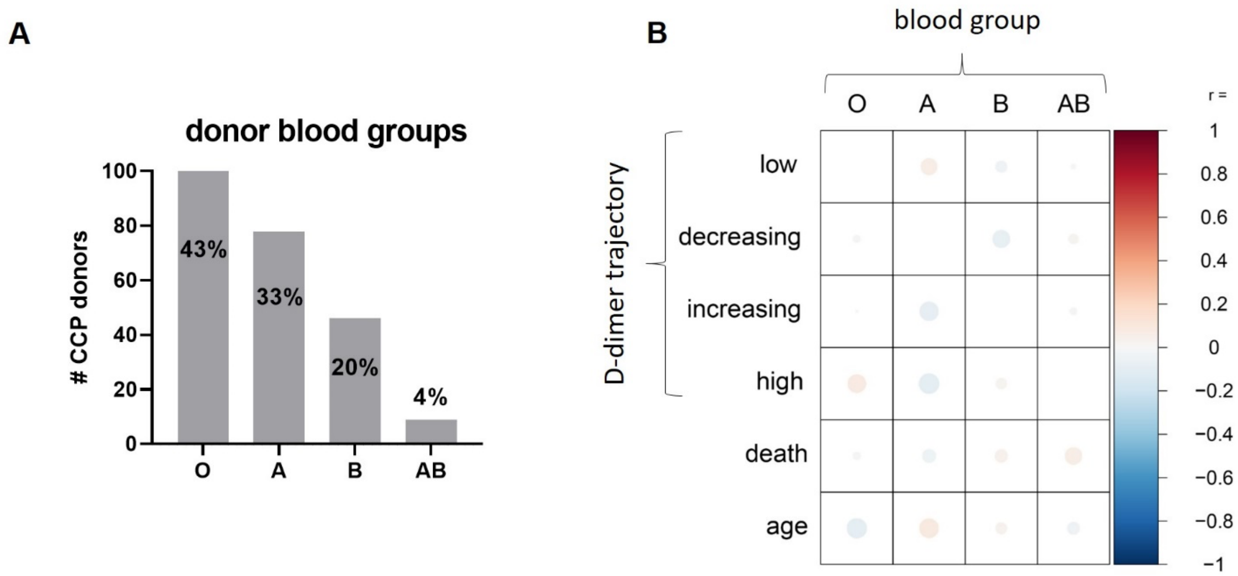

Supplemental Figure 2. Blood group of CCP donors does not correlate with any D-dimer trajectory, age, or mortality of CCP recipients. (A) Distribution of blood groups O, A, B, and AB among CCP donors (n = 233). (**B**) Blood group of donors does not correlate with the recipient's D-dimer trajectory, age or mortality. The correlogram is color-coded according to Spearman rank coefficient (r) between the respective pairwise variables.

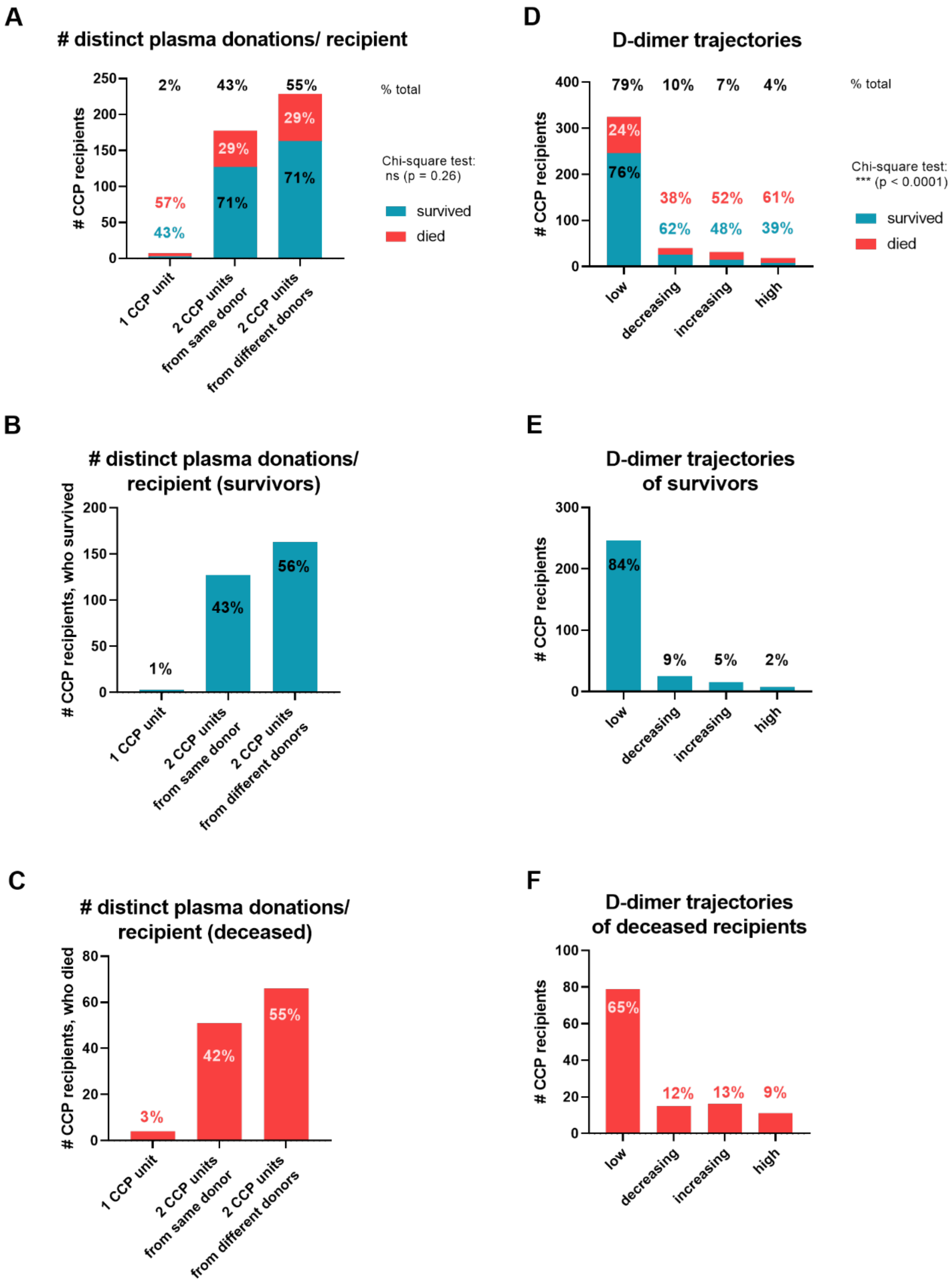

Supplemental Figure 3. Separate statistics for survived and deceased CCP recipients. Figure shows more detailed statistics related to Figure 2F and 2G. **(A)** Number of distinct plasma donations per recipient (survived and deceased), **(B)** only survivors and **(C)** only deceased patients. **(D)** D-dimer trajectories for all CCP recipients (survived and deceased), **(E)** only survivors and **(F)** only deceased patients.

Mean D-dimer score calculation for each donor

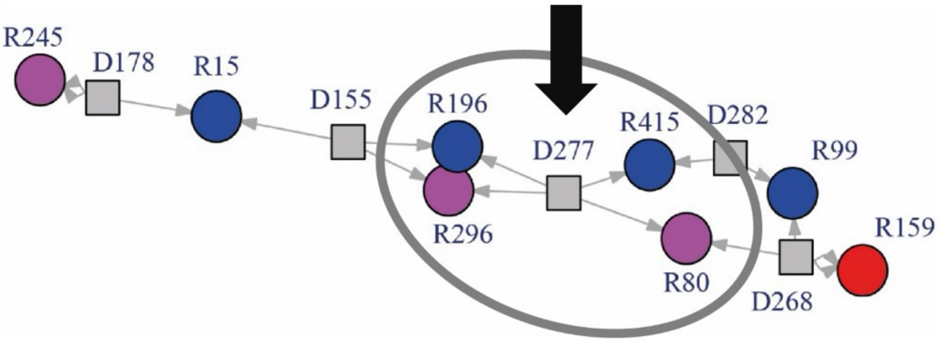

Each recipient with
- low or decreasing d-dimer trajectory = score of 1
- Increasing or high d-dimer trajectory = score of 2

Mean d-dimer score for each donor:
(Sum of all neighbor recipient scores) / (number of neighbor recipients)

Example for donor D277:
Its recipients are R196 (score 1), R296 (score 2), R415 (score 1) and R80 (score 2)
Mean d-dimer score: (1+2+1+2)/4 = 1.5

Supplemental Figure 4. Calculation of the mean D-dimer score for donors as risk factor assessment. A mean D-dimer score from the Donor-Recipient network was calculated to assess the “risk” associated with plasma from each of 304 CCP donors. Each CCP recipient was assigned a score of either 1 (if the D-dimer trajectory was persistently low or decreasing) or 2 (if the D-dimer trajectory was increasing or persistently high). Each donor's mean D-dimer network score was calculated by the sum of scores from each directly connected recipient divided by the number of directly connected recipients.

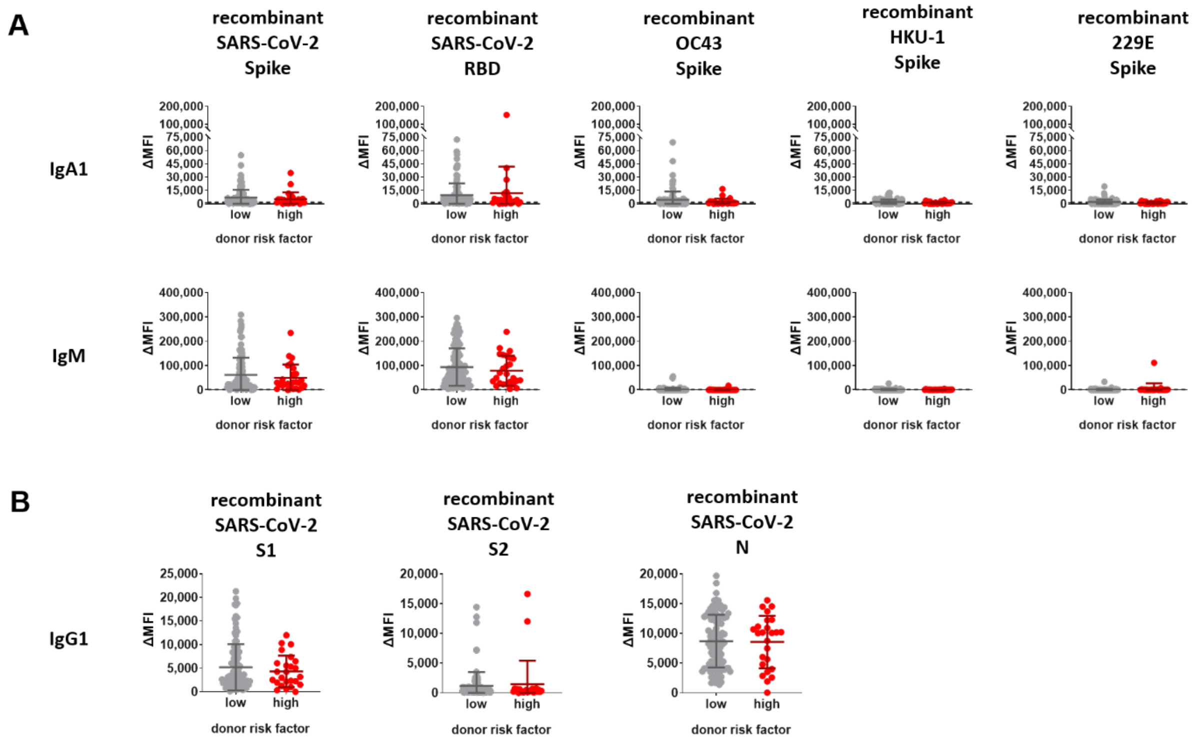

Supplemental Figure 5. Immune responses against the spike protein and subdomains of various coronavirus strains comparing "low" versus "high" risk CCP donors (**A**) Binding characteristics of IgA1 and IgM binding from donor CCP to recombinant proteins of SARS-CoV-2 spike, SARS-CoV-2 RBD, OC43 spike, HKU-1 spike and 229E spike using a Luminex bead-based multiplex assay comparing differences between antibodies associated with a high (D-dimer score <1.25) versus low score (D-dimer score >1.25). (**B**) IgG1 binding from CCP plasma was assessed against the S1, S2, and N (nucleocapsid) protein of SARS-CoV-2. Each experiment in (A) and (B) was measured in duplicate, showing the mean with SD for a total of n =135 CCP samples, including n = 109 low-risk and n = 26 high-risk samples.

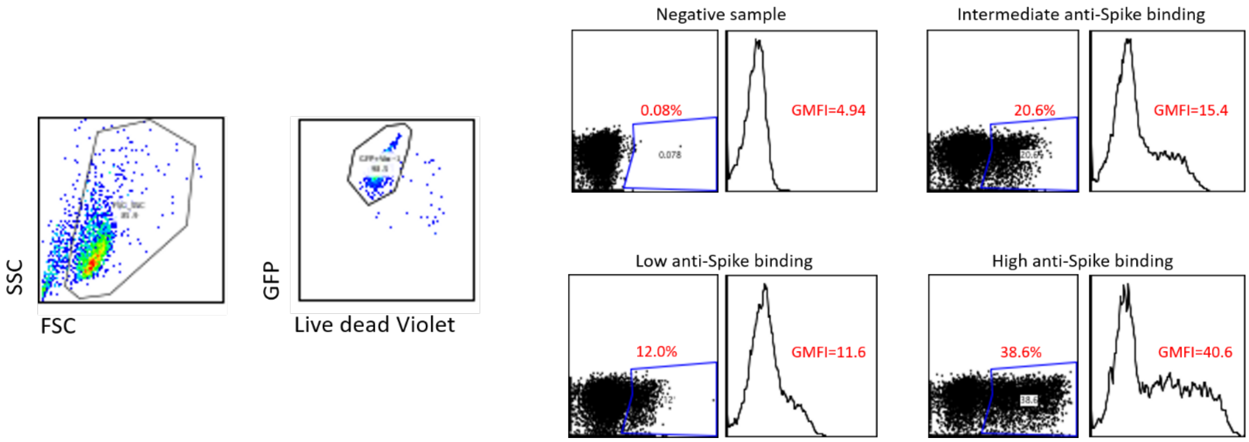

Supplemental Figure 6. Gating strategy for the antibody binding measured by an assay using cell-surface-expressed spike proteins. All cells were initially discriminated by side scatter (SSC) versus forward scatter (FSC) and dead cells were excluded by gating on the negative populations for LIVE/DEAD violet dead cell stain. Cell-surface binding of purified IgG from patients against the spike protein was compared to negative samples.

Supplemental Table 1: Adjustment for multiple comparisons of high- vs. low-risk CCP donors, showing p values, and adjusted p values (Benjamini-Hochberg). Asterisks indicate statistical significance (*p < 0.05; **p < 0.01; ***p < 0.005).

	p	adjusted p (Benjamini-Hochberg)
cell-surface binding_OC43	0.0149*	0.082
cell-surface_229E	0.5067	0.633
FcγR2a_SARS-CoV2	0.0029*	0.032*
FcγR2a_OC43	0.0651	0.143
FcγR2a_229E	0.106	0.194
FcγR3a_SARS-CoV2	0.0447*	0.143
FcγR3a_OC43	0.0593	0.143
FcγR3a_229E	0.5175	0.633
Auto-Abs_IFNα2	0.8266	0.827
Auto-Abs_IFNω	0.7157	0.787
Auto-Abs_IFNβ	0.4288	0.633

Clustering analysis of the donor-recipient network

We would like to determine whether or not the recipient status (L or H) is correlated with specific donors that were involved. Intuitively this is equivalent to determining whether or not H status individuals tend to have received plasma from the same subgroup of donors. To investigate this in a rigorous way, we will present the information about donor-recipient relationships in the form of a network, where two types of nodes (corresponding to donors and recipients) are connected by arrows (from each donor to all of their recipients). Figure S1 shows a small fragment of the resulting network. Here, the donors are colored purple, while recipients of L and H status are colored blue and pink, respectively. The numbers next to each node are unique identifiers. We can see, for example, that donor 135 donated to two recipients and donor 204 to three recipients. Recipient 8 obtained plasma from two distinct donors, while recipient 105 got both transfusions from the same donor.

Definition of a neighborhood and clustering analysis. We would like to assess whether H status individuals are more likely to have received plasma from the same donors. In terms of the network that would mean that H individuals are in some sense close to each other, or more precisely, are closer to each other than they would have been if they were distributed randomly and uniformly. To formalize the notion of closeness, we will define a neighborhood on this network in the following way: **For each recipient, i, its neighborhood B_i is a set of all the recipients that received transfusion from the donor(s) of individual i.**

For example, for recipient 253, their donors are 204 and 209, and the set of their recipients is $B_{253} = \{253, 178, 54, 70\}$, see panel (a) of figure S1, where individual 253 is indicated with a blue arrow and its neighborhood is encircled by a grey line. For recipient 54, their donors are 204 and 198, and the set of their recipients is $B_{54} = \{253, 178, 105, 54, 70\}$, see panel (b), where individual 54 is indicated with a red arrow and its neighborhood is encircled by a grey line.

Using this notion of a neighborhood, we can apply the clustering analysis that we used [1] in a different context. Let us denote the set of all L individuals as $\mathcal{L}$, and the set of all H individuals as $\mathcal{H}$. Further, we denote

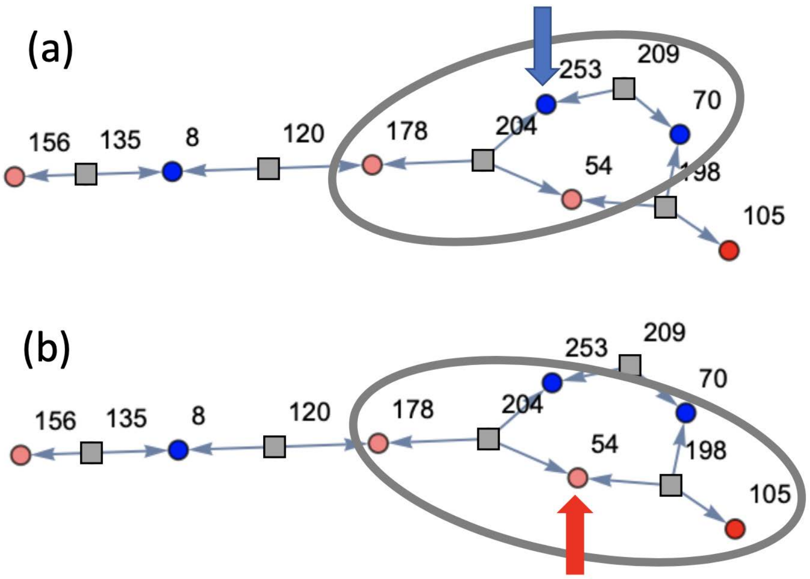

Figure S1: Definition of a neighborhood. Shown is a portion of the donor-recipient network, where gray squares denote donors, and blue (red) dots denote L (H) recipients. Arrows indicate transfusions, and numbers by each node are unique identifiers of donors and recipients. Grey ellipses indicate a neighborhood. (a) The neighborhood of recipient 253. (b) The neighborhood of recipient 54.

the total numbers of L and H recipients as N_L and N_H, respectively (that is, $|\mathcal{L}| = N_L$, $|\mathcal{H}| = N_H$).

For each L individual (say, individual i), we determine the number of its L neighbors, $L_i^{(L)}$, and the number of its H neighbors, $H_i^{(L)}$ (here, the superscript (L) indicates the fact that we are considering neighbors of an L individual). For example, for recipient 253 (figure S1(a)), we have $L_{253}^{(L)} = 1, H_{253}^{(L)} = 2$.

Similarly, for each H individual (say, individual j), we determine the number of its L neighbors, $L_j^{(H)}$, and the number of its H neighbors, $H_j^{(H)}$ (here, the superscript (H) indicates the fact that we are considering neighbors of an H individual). For example, for recipient 54 (figure S1(b)), we have $L_{54}^{(H)} = 2, H_{54}^{(H)} = 2$.

For all L recipients, we can calculate the mean number of L and H neigh-

bors:

$$L^{(L)} = \frac{1}{N_L} \sum_{i \in \mathcal{L}} L_i^{(L)}, \quad H^{(L)} = \frac{1}{N_L} \sum_{i \in \mathcal{L}} H_i^{(L)}.$$

Similarly, for all H recipients, we calculate the mean number of L and H neighbors:

$$L^{(H)} = \frac{1}{N_H} \sum_{i \in \mathcal{H}} L_i^{(H)}, \quad H^{(H)} = \frac{1}{N_H} \sum_{i \in \mathcal{H}} H_i^{(H)}.$$

The imbalance measure. The mean numbers of L and H recipients found in each of the neighborhoods are expected to be correlated with the total number of L and H recipients. In fact, if the labels H and L are given completely randomly, the quantities above will be proportional to the total numbers of L and H individuals. Therefore, in order to detect a deviation from randomness, we can study the normalized quantities, such as $H^{(H)}/(N_H - 1)$ and $L^{(H)}/N_L$. Note that $H^{(H)}$ is divided by $N_H - 1$, not by N_H, because the H individual whose H neighbors are included in the summation, does not enter the total count. For a random placement of N_L individuals of type L and N_H individuals of type H on a network, these normalized quantities have the same distribution and are equal to each other, if averaged over a large number of trials. Therefore, a convenient measure of prevalence of H individuals in the proximity of each other is given by the imbalance measure,

$$I(H) = \frac{H^{(H)}/(N_H - 1)}{H^{(H)}/(N_H - 1) + L^{(H)}/N_L}.$$

This quantity considers the L and H counts in the neighborhood of H recipients, and measures their deviation from being equal. The prevalence of L individuals in the proximity of each other can be defined similarly.

If $I(H)$ is greater than $1/2$, this may signal the presence of preferential clustering of H individuals near each other (that is, near the same donors). The data show that $I(H) = 0.729 > 1/2$, that is, H recipients tend to cluster together.

To assess the statistical significance of clustering behavior in the dataset, we created a large number of artificial donor-recipient networks, where the relative "locations" of recipients and donors were exactly the same, but the recipient status was randomized (while preserving the total number of L and H individuals). We created $10,000$ of such artificial random datasets, and calculated the value $I(H)$ for each of them. The results are summarized in

the form of a histogram in the main text, figure 2i. Calculating the fraction of random realizations whose values for $I(H)$ were larger than the ones for the real-life data set, we obtained an estimate for the p-value, $p \approx 7 \times 10^{-4}$. Therefore, the hypothesis that the recipient status is independent of the donor can be rejected at the 0.01 level.

Similar analysis was performed to incorporate recipients' mortality status, rather than their D-dimer trajectory status. In particular, instead of status values L and H, we used information on whether or not the recipient survived (values S for survivors and N for non-survivors). We then calculated the quantity $I(N)$ which showed whether or not non-survivors tended to cluster together. We obtained $I(N) = 0.51$, which, although slightly larger than 1/2, did not deviate from 1/2 significantly ($p > 0.1$).

References

[1] Kenneth M Law, Natalia L Komarova, Alice W Yewdall, Rebecca K Lee, Olga L Herrera, Dominik Wodarz, and Benjamin K Chen. In vivo HIV-1 cell-to-cell transmission promotes multicopy micro-compartmentalized infection. *Cell reports*, 15(12):2771–2783, 2016.
